# PRIMA-1 increases cisplatin sensitivity in chemoresistant ovarian cancer cells with p53 mutation: a requirement for Akt down-regulation

**DOI:** 10.1186/1757-2215-6-7

**Published:** 2013-01-26

**Authors:** Noriko Kobayashi, Mohammadreza Abedini, Noriaki Sakuragi, Benjamin K Tsang

**Affiliations:** 1 Department of Obstetrics & Gynecology and Cellular & Molecular Medicine, University of Ottawa; Chronic Disease Program, Ottawa Hospital Research Institute, Ottawa K1H 8L6, Canada; 2 Department of Gynecology, Hokkaido University Graduate School of Medicine and School of Medicine, Sapporo, Japan; 3 Cellular and Molecular Medicine Research Center, Department of Physiology and Pharmacology, Birjand University of Medical Sciences, Birjand, Iran; 4 World Class University (WCU) Biomodulation Major, Department of Agricultural Biotechnology, College of Agriculture and Life Sciences, Seoul National University, Seoul, Republic of Korea

**Keywords:** PRIMA-1, Chemoresistance, Ovarian cancer, Akt, p53, Cisplatin

## Abstract

**Background:**

Since ovarian cancer is associated with high frequency of p53 mutation, the availability of p53 reactivation and induction of massive apoptosis (PRIMA-1) offers a possible new therapeutic strategy for overcoming this devastating disease. Although Akt activation is believed to be a determinant in chemoresistance in ovarian cancer, whether Akt plays a role in regulating the effectiveness of PRIMA-1 in sensitizing chemoresistant ovarian cancer cells with p53 mutation to cisplatin (CDDP), remains to be determined.

**Methods:**

In the present studies, we examined the influence of Akt down-regulation following dominant-negative (DN-Akt) expression on the ability of PRIMA-1 (0–10 μM) to facilitate CDDP (0–10 μM)-induced apoptosis in p53-mutated chemoresistant ovarian cancer cells (A2780cp).

**Results:**

Apoptosis rate was significantly higher at the combined treatment of low PRIMA-1 concentrations (0.156 - 0.938 μM) plus CDDP (10 μM) in the DN-Akt groups than control (p<0.001). Apoptosis in cells treated with PRIMA-1 (0.156 μM) and CDDP treatment (10 μM) was significantly suppressed by p53-siRNA. PRIMA-1 increased phospho-p53 (Ser15) content in Akt down-regulated cells treated with CDDP.

**Conclusions:**

These results demonstrate that PRIMA-1 can sensitize chemoresistant ovarian cancer cells with p53 mutation to CDDP when Akt is down-regulated, and the action of PRIMA-1 is associated with p53 activation. Our findings raise the possibility that PRIMA-1 may be useful candidate for adjuvant therapy with CDDP in chemoresistant ovarian cancer with p53 mutation when Akt is down-regulated.

## Introduction

Ovarian cancer is the most lethal gynecological malignancies. Currently, the preferred treatment for ovarian cancer is combination chemotherapy, usually with a platinum based drug (e.g. CDDP or carboplatin), together with surgical debulking. The effectiveness of many of the chemotherapeutic agents in human cancer is highly dependent on the ability of the cancer cells to undergo drug-induced apoptosis. The development of chemoresistance is a major clinical problem for successful treatment in human ovarian cancer.

The tumor suppressor p53 inhibits tumor growth primarily by induction of apoptosis through mechanisms which are transcription-dependent [1,2] and –independent [3-5]. p53 binds to a specific DNA sequence and transactivates target genes leading to cell cycle arrest and/or apoptosis. p53 dependent apoptosis is an important determining factor on the efficacy of chemotherapy, as tumors with p53 mutation are often more resistant to chemotherapeutic agents compared to those harboring wild-type p53 (wt-p53) [6,7]. Mutations in p53 occur in nearly half of human ovarian tumors. A majority being missense mutations in the DNA-binding core domain, thus resulting in deficient DNA binding [8].

Akt is a serine/threonine kinase activated by growth factors and cytokines in a phosphatidylinositol-3-OH-kinase (PI3K)-dependent manner [9,10]*.* Akt is implicated in cell proliferation and survival and is a key determinant of CDDP resistance in ovarian cancer cells which are p53 dependent [5]. We have previously demonstrated that Xiap, Akt and p53 interact in the regulation of chemosensitivity in ovarian cancer cells [2]. The PI3K-Akt pathway is over-expressed or activated in chemoresistant ovarian cancer cells and Akt down-regulation sensitizes chemoresistant wt-p53 cells to CDDP-induced apoptosis [2,11]. The latter response, however, is not evident in mutant-p53 cells unless reconstituted with wt-p53.

PRIMA-1, a low molecular weight compound, is more effective in inducing apoptosis in mutant-p53 cells than the wt-p53 cells and has noticeable anti-tumor activity *in vitro* and *in vivo *[12]*.* The sensitivity of PRIMA-1 was related to mutant p53 expression levels [13]. It is capable to induce apoptosis in human tumor cells through restoring the transcriptional function to mutant-p53 [14]. PRIMA-1 and the structural analog PRIMA-1 MET, also named APR-246, reactivate mutant p53 through covalent binding to the core domain and induce apoptosis in tumor cells. Its anti-tumor effect does not appear to be due to general toxicity [14]. Although PRIMA-1 is capable of restoring chemosensitivity in mutant-p53 cells, whether it acts synergistically with CDDP to inhibit proliferation of mutant-p53 ovarian cancer cells is unclear. Moreover, whether Akt plays a role in regulating the effectiveness of PRIMA-1 in sensitizing chemoresistant mutant-p53 ovarian cancer cells to CDDP, remains to be determined.

In the present studies, we have investigated the role of Akt in this regards and demonstrated that Akt down-regulation induce significant apoptosis in combination treatment of PRIMA-1 and CDDP in chemoresistant ovarian cancer cells carrying p53 mutation.

## Materials and methods

### Reagents

Cells were cultured at 37°C with 5% CO2 in DMEM (Dulbecco’s modified Eagle’s medium)/F12 (Invitrogen Inc., Burlington, ON, Canada). Medium was supplemented with 10% fetal bovine serum (FBS), streptomycin (100 ***μ***g/mL), penicillin (100U/mL), and fungizone (0.625 μg/mL). PRIMA-1 was purchased from Calbiochem, Inc. (San Diego, CA, USA). *Cis-*diaminedichloroplatinum (CDDP) and Hoechst 33258 were supplied by Sigma (Oakville, ON, Canada). Adenoviral dominant-negative Akt (DN-Akt) was a gemerous gift from Dr. Kenneth Walsh (Cardiovascular Research, St. Elizabeth’s Medical Centre, Boston, MA). Adenoviral LacZ was synthesized at the Neuroscience Research Institute, University of Ottawa (Ottawa, ON, Canada). Small inhibitory RNA (siRNA) to p53, scrambled sequence siRNA (control) and Mouse monoclonal anti-phospho-p53 (Ser15) were from Cell Signaling Technology Inc. (Beverly, MA, USA). Mouse monoclonal anti-glyceraldehyde phosphate dehydrogenase (GAPDH) was from Abcam (Cambridge, MA, USA).

### Cell culture, adenoviral infection and treatment of PRIMA-1 and CDDP

Chemoresistant ovarian cancer cells (A2780cp: p53-mutant cell line) were cultured and treated as reported previously [2]. Cells were plated into 60 mm dishes in DMEM/F12 and infected with adenoviral DN-Akt construct (MOI = 40) for 48 h. Infection with adenoviral LacZ served as control and was used to normalize the total dose of adenovirus be same in each treatment group. Cells were treated with PRIMA-1 (0–10 μM) for 8 hours, and then harvested at 24 hours following CDDP treatment (0–10 μM). All CDDP treatment was performed in serum-free media.

### Assessment of apoptosis

At the end of treatment period, cells attached to the growth surface were harvested by trypsin treatment. Floating and attached cells were then pooled and centrifuged, and the pellet were resuspended in phosphate buffered formalin (10%) containing Hoechst 33258 (12.5 ng/ml). Cells were spotted onto slides and changes in nuclear morphology were observed using a Zeiss fluorescence microscope (magnification 400X), as previously reported [15,16]. A minimum of 200 cells with typical apoptotic nuclear morphology (nuclear shrinkage, condensation and fragmentation) were counted in each treatment group from randomly selected fields and expressed as the percentage of total cells [3,17]. The counter was “blinded” to sample identity to avoid experimental bias.

### Transfection with p53-siRNA

After 12–18 hours of plating, cells were infected with adenoviral DN-Akt (MOI = 40; 48 h). To determine if the action of PRIMA-1 was mediated by p53, the cells were, transfected with p53-specific or control siRNA (50 nM) 24 h after the infection and then treated with PRIMA-1 (0.156 μM) for 8 h. The cells were harvested at 24 h after CDDP treatment (10 μM). p53 down-regulation was confirmed by Western blot analysis [3,4,15,17].

### Protein extraction and Western blotting

Protein extraction and Western blotting were performed as described previously [2]. Membranes were incubated overnight at 4°C with primary anti-p53 (1:1000), anti-GAPDH (1:2,000), and subsequently detected with horseradish peroxidase-conjugated goat IgG raised against the corresponding species. Peroxidase activity was visualized with an enhanced chemiluminescence (ECL) kit (Amersham Biosciences, Piscataway, NJ). Signal intensity was determined densitometrically using Scion Image software, version 4.02, from Scion Corporation (Frederick, MD, USA).

### Statistical analyses

All results are expressed as mean ±SEM of at least three independent experiments. Data were analyzed by two-way ANOVA and the differences between multiple experimental groups determined by Bonferoni post-hoc tests (PRISM software version 3.0, GrahPad, San Diego, CA). Statistical significance was inferred at P<0.05.

## Results

### PRIMA-1 together with Akt down-regulation sensitizes chemoresistant ovarian cancer cells with mutant-p53 to CDDP in vitro

**Figure 1:**
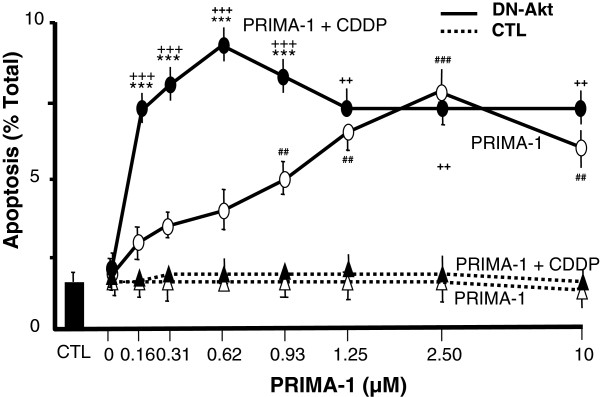
**The effect of different concentration of PRIMA-1 on CDDP-induced apoptosis in chemoresistant p53-mutant ovarian cancer cells with/without Akt down-regulation.** Apoptosis rate was evaluated with difference concentration of PRIMA-1 (0–10 μM) plus CDDP (0 or 10 μM) using adenoviral DN-Akt (^**_____**^) or LacZ (^**……**.^; as control). *** P<0.001; PRIMA-1+CDDP & DN-Akt vs. PRIMA-1 & DN-Akt; ^+++^ P<0.001, ^++^ P<0.01 ; PRIMA-1+CDDP & DN-Akt vs. PRIMA-1+CDDP & CTL; ^###^ P<0.001, ^##^ P<0.01 ; PRIMA-1+CDDP & DN-Akt vs. PRIMA-1 & CTL. Results are expressed as mean ± SEM of three independent experiments

Tumor suppressive p53 is required for CDDP sensisitivity [1,2]. P53 mutation is often associated with chemoresistance in ovarian cancer [18]. We have previously demonstrated that CDDP is unable to induce apoptosis in p53-mutated ovarian cancer cells unless reconstituted with wt-p53 and Akt function down-regulated. To investigate whether PRIMA-1 increases CDDP sensitivity in mutant-p53 chemoresistant ovarian cancer cells and if its action is depending on Akt down-regulation, A2780cp cells were infected with adenoviral DN-Akt (MOI = 40; LacZ as control) for 48 h, treated with PRIMA-1 (0–10 μM; 8 h), and then harvested after CDDP treatment (0–10 μM; 24 h). As shown in Figure 1, in the presence of CDDP and DN-Akt, apoptosis rate was significantly higher in PRIMA-1 (0.156 - 10 μM; p<0.01) than LacZ control. While PRIMA-1 alone was ineffective in the LacZ control groups, it significantly induced apoptosis in the DN-Akt groups in a concentration-dependent manner (0.938 - 10 μM; p<0.01). Apoptosis was significantly induced in the low concentration of PRIMA-1 (0.156 - 0.938 μM: p<0.01) plus CDDP groups compared to PRIMA-1 alone group with DN-Akt (p<0.001), this response was not evident at higher concentrations (1.25 – 10 μM; p > 0.05). There was no difference in apoptosis rate between PRIMA-1 alone groups and PRIMA-1 plus CDDP groups without DN-Akt.

**Figure 2:**
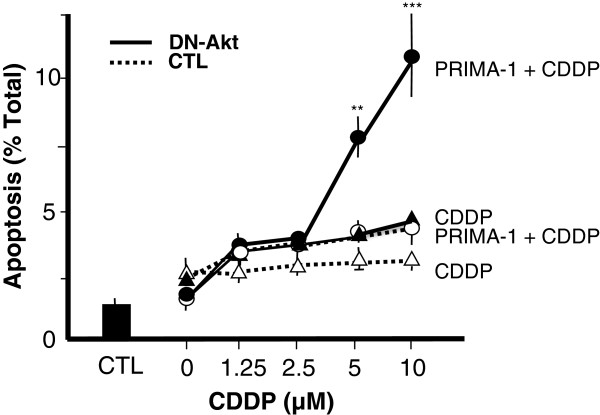
**The influence of different CDDP concentration on the PRIMA-induced apoptosis following Akt down-regulation.** Apoptosis rate was analyzed with different concentration of CDDP (0–10 μM) plus PRIMA-1 (0 or 0.625 μM) which was strong enough to induce apoptosis in the combined treatment of CDDP in the absence (^**…….**^) and presence (^**_____**^) of DN-Akt. ^###^ P<0.001, ^##^ P<0.01; PRIMA-1+CDDP & DN-Akt vs. PRIMA-1 & CTL. Results are expressed as mean ± SEM of three independent experiments

To further examine the role of PRIMA-1 in the regulation of CDDP sensitivity, the above experiment carried out with different concentration of CDDP (0–10 μM) and PRIMA-1 (0.625 μM) which was strong enough to induce apoptosis in the combined treatment of CDDP in the absence and presence of DN-Akt. While CDDP was unable to induce apoptosis with LacZ control, PRIMA-1 and/or DN-Akt groups, it induces cell death in the presence of both PRIMA-1 and DN-Akt group in a concentration dependent manner (Figure 2). Apoptosis was significantly higher in the DN-Akt groups in a CDDP concentration (5–10 μM) with maximal response observable at 0.625 μM PRIMA-1 and 10 μM CDDP.

### p53 - specific action of PRIMA-1

**Figure 3:**
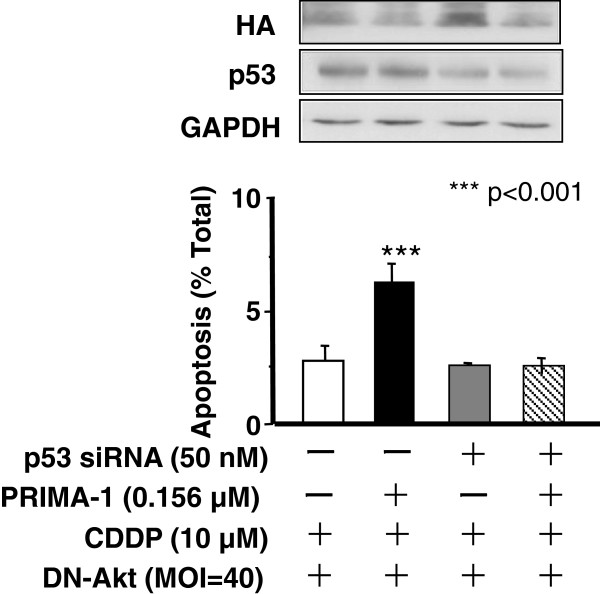
**PRIMA-1-facilitated CDDP-induced apoptosis in Akt down-regulated chemoresistant p53-mutant ovarian cancer cells is mediated by p53.** Akt function was down-regulated by adenoviral DN-Akt infection (MOI = 40). Apoptotic rate was evaluated to determione whether p53 is involved in the synergistic effect of PRIMA-1 and CDDP following transfection with p53 or control siRNA. *** p<0.001 vs CDDP & DN-Akt; Results are expressed as mean ± SEM of three independent experiments

We next elucidated whether p53 is involved in the synergistic effect of PRIMA-1 and CDDP with DN-Akt (Figure 3). A2780cp cells were infected with adenovirus containing DN-Akt (MOI = 40; 48 h), transfected with p53 or control siRNA, treated with least concentration of PRIMA-1 (0–0.156 μM; able to induce significant apoptosis in above experiments; 8 h), and harvested at 24 h following CDDP treatment (10 μM; 24 h). As demonstrated in Figure 3, PRIMA-1 and CDDP were unable to sensitize the cells neither in the presence of p53 nor control siRNA. However, the combination groups of PRIMA-1 and CDDP with DN-Akt dramatically induces apoptosis in the cells transfected with control siRNA, a response which was significantly suppressed in the group with p53-siRNA.

### The action of PRIMA-1 is associated with p53 activation in vitro

**Figure 4:**
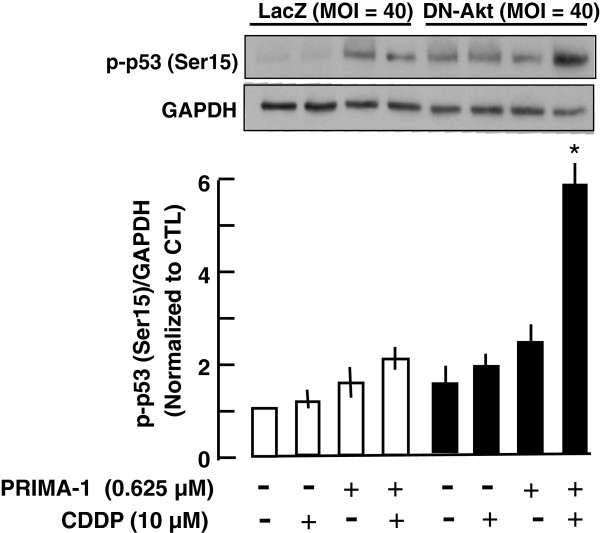
**PRIMA-1 increases p-p53 content in chemoresistant p53-mutant ovarian cancer cells treated with CDDP when Akt was down-regulated.** Changes in p-p53 (Ser15) content following PRIMA-1 and/or CDDP treatment with/without Akt down-regulation were assessed by Western blot. Results are normalized against GAPDH and expressed as fold of control. * p<0.05 vs CDDP & DN-Akt; Values are mean ± SEM of three independent experiments

PRIMA-1 sensitizes the effect of CDDP when Akt function is down-regulated in p53-mutant ovarian cancer cells. Although our data suggests that p53 is required for this effect, the mechanism involved is unclear. To determine whether the action of PRIMA-1 is mediated through p53 phosphorylation and thus its activation, phospho-p53 (Ser15) content in A2780cp cell extracts from the above experiments were determined by Western blot. As shown in Figure 4, p53 phosphorylation was not evident in LacZ group in the absence and presence of CDDP, although this response was detected with PRIMA-1 and DN-Akt alone and in the presence of CDDP. Down-regulation of Akt markedly enhanced this response, activated p53 and sensitized the cells in the induction of apoptosis by the combined treatment with PRIMA-1 and CDDP. PRIMA-1 increased phospho-p53 (Ser15) content in Akt down-regulated cells treated with CDDP, suggesting that the action of PRIMA-1 in facilitating CDDP-induced apoptosis in p53 mutant chemoresistant ovarian cancer cells may in part be mediated through increased p53 phosphorylation at Ser15 (Figure 4).

## Discussion

In the present study, we have shown that PRIMA-1 can sensitize chemoresistant ovarian cancer cells with p53 mutation to CDDP when Akt function is down-regulated. Our data also suggest that the action of PRIMA-1 is associated with p53 phosphorylation and activation. The mechanism by which PRIMA-1 sensitizes mutant p53 and induces apoptosis has not been well elucidated. Whether PRIMA-1 binds directly to mutant p53 or it acts through indirect mechanisms remains an opened question. p53 binds to a specific DNA sequence and transactivates target genes involved in the regulation of cell cycle arrest and apoptosis. Tumor cells containing wt-p53 are usually more chemosensitive than those bearing mutant p53. We have previously shown that while CDDP up-regulates p53 in CDDP-sensitive wt-p53 cells (OV2008), but not its resistant wt-p53 variant (C13*) *in vitro*, suggesting that regulation of p53 content/function by CDDP may be an important determinant of sensitivity [2].

PRIMA-1 synergizes with chemotherapeutic drugs to induce tumor cell apoptosis [12,19-21]. PRIMA-1 restores wild-type confirmation to mutant p53 by binding to the core and induces apoptosis in human tumor cells. Whereas wt-p53 is rapidly degraded by MDM2 in normal cells, the mutant p53 protein fails to undergo degradation in tumor cells and accumulates extensively [22]. Several studies have demonstrated that PRIMA-1 is able to restore the sequence-specific DNA-binding and to transactivate some mutant p53 proteins *in vitro* and to induce apoptosis *in vivo *[12,23-25]. p53 activates many genes involved in cell cycle arrest and apoptosis, mainly through its transcription-dependent activity [26,27]. It is essential that p53 reactivation in tumor cells trigger apoptosis rather than cell arrest, as the therapeutic goal is to kill the tumor cells. Heat shock protein 90 is a candidate target for p53 mutation reactivation by PRIMA-1 in breast cancer cells [28]. Some data have indicated that treatment with PRIMA-1 leads to upregulation of at least some of p53 target genes; for example, Bax and Noxa but not c-Jun-NH2-kinase (JNK) signaling [29,30]. On the other hand, Li et al. reported that JNK pathway plays an important role on PRIMA-1-induced apoptosis [31]. Others have shown that PRIMA-1 is capable of inducing apoptosis in a transcription independent manner [32] or even mutant p53-independent [33]. It has also been reported that PRIMA-1 induces activation of caspase-2, caspase-3 and caspase-9, consistent with induction of apoptosis via the mitochondrial pathway [25]. Microarray analysis revealed that PRIMA-1 induces a limited set of genes in a mutant p53-dependent manner, followed by ER stress [34].

Akt activation promotes cell survival, suppresses apoptotic death and confers resistance of ovarian cancer cells to CDDP-induced apoptosis [2,11,35]. Over-expression/activation of the PI3K-Akt pathway is commonly observed in ovarian cancer [36,37]. However, precisely how Akt controls p53 activation is still unclear. Activation of Akt promotes the entry of MDM2 into the nucleus and its interaction with the tumor suppressor protein p53. Binding of MDM2 to p53 inhibits the transcriptional activity of p53 and targets it for proteasomal degradation [38]. We previously demonstrated that activated Akt is an important regulator of both X-linked inhibitor of apoptosis protein (Xiap) and p53 levels after CDDP challenge and that p53 mutational status is a determinant of Akt-mediated chemoresistance [2,11]. Inhibition of Akt activity facilitated the CDDP-induced mitochondrial release and nuclear accumulation of apoptosis-inducing factor (AIF)-dependent, CDDP-induced apoptosis [39]. Activation of Akt confers resistance by blocking p53-mediated transactivation and p53 phosphorylation [1]. In the present study, suppression of Akt sensitized chemoresistant cells to CDDP in a p53-dependent manner, suggesting a functional link between Akt-mediated chemoresistance and p53.

Recent data demonstrated that p53 is essential for CDDP-induced apoptosis in human ovarian cancer cells, and that p53-mediated apoptosis is dependent on the phosphorylation of several N-terminal residues, including Ser15, Ser20 and Ser37 [40-42]. Our previous data suggested that this is mediated, at least in part, through the phosphorylation of p53 on Ser15 and Ser20 [1]. As Ser15 phosphorylation affects p53 stability [43], phosphorylation of p53 at serine15 by PRIMA-1 seems to be involved in the reactivation of p53 transcriptional function. Mutation of Ser15 to Ala significantly attenuates p53-mediated apoptosis [42]. Our data show that down-regulation of Akt sensitizes the cells to CDDP-induced apoptosis and p-p53 at Ser15 is associated in the PRIMA-1-CDDP interaction, suggesting that Ser15 phosphorylation is needed for its function and apoptosis. This also suggests that CDDP induces p53 phosphorylation on Ser15 residue, which absent in chemoresistant cells and is required for CDDP-induced apoptosis. Akt efficiently blocks this processes, thereby conferring resistance to CDDP-induced apoptosis. In addition, while Akt modulates other p53-dependent cellular events, including the down-regulation of FLIP, our evidence suggests that effective p53 phosphorylation and activation is essential for CDDP-induced apoptosis. Thus, it will be of interest to study the effects of chemotherapy on total and phospho-p53 in human ovarian tumors. Moreover, since Akt attenuates both processes, it is important to study the relationship between activation/overexpression of Akt in ovarian tumors and sensitivity to CDDP.

In summary, inhibition of Akt activity may represent a novel therapeutic approach to the combined treatment of PRIMA-1 and CDDP. Further examination of the role and regulation of Akt in the PRIMA-1-CDDP interaction in the ovarian tumour xenograft might provide novel insights into a possible new therapy for chemoresistant ovarian cancer.

## Competing interests

The authors declare no conflict of interests.

## Authors’ contributions

NK carried out the the experiments and drafted the manuscript. MRA participated in the design of the studies, reviewed the data and revised the manuscript. NS and BKT provided input in the project, reviewed the data and the manuscript. All authors read and approved the final manuscript.
